# Supplementary material for: Sound symbolism in Japanese names: Machine learning approaches to gender classification
Source: PLoS One. 2024 Mar 11;19(3):e0297440. doi: 10.1371/journal.pone.0297440 (PMC10927153; doi:10.1371/journal.pone.0297440)
Supplement: Attachment — Submitted filename: Response to Reviewers.pdf [file pone.0297440.s001.pdf]

Ngai Chun Hau

East Asian Languages and Cultures Department

Indiana University, Bloomington

12th October, 2023

Dr. Søren Wichmann

Academic Editor

PLOS ONE

Dear Dr. Wichmann,

We would like to express our sincere appreciation for the opportunity to revise our manuscript titled "Sound Symbolism in Japanese Names: Machine Learning Approaches to Gender Classification" in response to the valuable feedback provided by the editor. We have carefully considered your comments and suggestions, and we believe that the revised manuscript now adequately addresses the concerns raised prior the review process.

We have made substantial revisions to the manuscript to improve to adherence to PLOS ONE's formatting criteria. Specifically, we have addressed the following key points:

- Presentation and Stylistic Issues: We have thoroughly revised the manuscript to rectify the typos, grammatical errors, stylistic problems, and references pointed out by the editor. We have carefully proofread the entire manuscript, starting from the abstract to the references section, to ensure its accuracy and readability.
- Format Requirements: We have carefully followed PLOS ONE's style requirements, including those for file naming. We have reviewed the PLOS ONE style templates provided at the URLs https://journals.plos.org/plosone/s/file?id=wjVg/PLOSOne_formatting_sample_main_body.pdf and https://journals.plos.org/plosone/s/file?id=ba62/PLOSOne_formatting_sample_title_authors_affiliations.pdf, and we have adjusted the format of our manuscript accordingly.
- Code Sharing: As per PLOS ONE's guidelines on code sharing, we could assure you that we will make the osf repository publicly available once the manuscript is accepted. Currently the code has been uploaded to the public repository https://osf.io/yrx4u/?view_only=d7fc5ef8b4ab449d8aaba591b18fdfc9
- Grant Information and Financial Disclosure: We apologize for the previous discrepancies in the 'Funding Information' and 'Financial Disclosure' sections. We have revised the manuscript to provide accurate grant numbers for the awards we received for our study in the 'Funding Information' section. Furthermore, we have included an updated financial disclosure statement in our cover letter to reflect the necessary changes.

- Tables: We have included new copies of Tables 2, 4, 5, and 7 in the manuscript, following the formatting guidelines provided by PLOS ONE. Additionally, we discovered that table 8 and 9 were a mistake due an error in table and figure naming. There was no table 8 and 9 to begin with. In fact, these tables are table 6 and 7. Table 6 and 7 presented the results obtained from Random Forest and XGBoost which could be found in page 14 and 15 respectively.
- Furthermore, we have attached a photo of Figure 1 in response to the request for additional figure files. This addition provides further clarity and support to our findings.

We would like to emphasize that we have taken great care to address all the comments and suggestions provided by the editor. We believe that the revised manuscript now meets PLOS ONE's publication criteria and contributes significantly to the field of sound symbolism in Japanese names.

Once again, we sincerely appreciate the valuable feedback provided by the editor and the editor. We are confident that the revised manuscript will be well-received by the scientific community. Thank you for your time and consideration.

Sincerely,

Ngai Chun Hau

Indiana University, Bloomington

chngai@iu.edu
